# Registration of Births, Marriages, and Deaths

**Published:** 1853-08

**Authors:** 


*Registration of Births, Marriages, and Deaths.* By FRANKLIN TUTHILL, M. D. (Transactions N. Y. State Med Society.)

This is an able exposition of the necessity of such a law. No specific plan is proposed for the details of its working. We have in one corner of our cerebrum, a plan wherein we have much faith; and one of these days we shall enunciate it. S. B. H.

